# Extraintestinal Manifestations and Other Comorbidities in Ulcerative Colitis and Crohn Disease: A Danish Nationwide Registry Study 2003–2016

**DOI:** 10.1093/crocol/otaa070

**Published:** 2020-08-25

**Authors:** Kasper Vadstrup, Sarah Alulis, Andras Borsi, Tine Rikke Jørgensen, Agnete Nielsen, Pia Munkholm, Niels Qvist

**Affiliations:** 1 Janssen Immunology, Janssen-Cilag, Birkerød, Denmark; 2 Janssen Immunology, Janssen-Cilag, High Wycombe, UK; 3 Department of Market Access and Health Economy, Incentive, Holte, Denmark; 4 Gastroenterology Department, North Zealand University Hospital, Capital Region, Frederikssund, Denmark; 5 Surgical Department A and IBD Care, Odense University Hospital, Odense, Denmark

**Keywords:** epidemiology, extraintestinal manifestations, inflammatory bowel disease

## Abstract

**Background:**

Extraintestinal manifestations (EIMs) in inflammatory bowel disease (IBD) may be a frequent complication to an underlying abnormal immune response. This study investigated the occurrence of EIMs in Crohn disease (CD) and ulcerative colitis (UC) patients using population-based data in Denmark from 2003 to 2016.

**Methods:**

In this national registry-based study, incident CD and UC patients between 2003 and 2015 were matched on age and gender with non-IBD controls and followed until 2016. The selected EIMs for this study included 51 different diagnoses divided into biological systems of diseases, which were tested for differences in the timing and occurrence of EIMs.

**Results:**

The study cohort included 10,302 patients with CD and 22,144 patients with UC. The highest risk of patients experiencing EIM/comorbidities for the first time before their IBD diagnosis was in the skin and intestinal tract systems. For CD, the odds ratio of having an EIM before or after IBD diagnosis, as compared with controls, was significant in the skin, intestinal tract, hepatopancreatobiliary, musculoskeletal, ocular, renal, and respiratory systems. For UC, the risks were similar before and after UC diagnosis, apart from the nervous system where the odds ratio was significantly higher before the diagnosis of UC, and significantly lower after diagnosis for diseases in the ocular system.

**Conclusions:**

EIMs in CD and UC patients may also precede their IBD diagnosis. These findings may indicate a significant diagnostic delay of CD and UC, and the occurrence of known EIMs should prompt physicians to look for patients possibly having underlying IBD.

## INTRODUCTION

Inflammatory bowel disease (IBD) is a chronic inflammatory state of the gastrointestinal tract with an increasing incidence globally. IBD is classified into 2 main clinical phenotypes, Crohn disease (CD) and ulcerative colitis (UC). The exact pathogenesis of IBD is unknown, but factors such as abnormal gut microbiota, immune response dysregulation, environmental exposures, and gene variants may play a role. Patients with UC typically have mucosal inflammation starting in the rectum that can extend continuously to proximal segments of the colon, while CD is transmural and can affect all segments of the gastrointestinal tract, the most common being the terminal ileum and colon.^1,2^

IBD is associated with a variety of extraintestinal manifestations (EIMs). The reported frequency varies from 14% to 47%, and accounts for the majority of comorbidities in IBD, and in many cases the EIMs are more bothersome for the patient than the IBD itself.^3–6^ EIMs may present prior to the diagnosis of IBD or after.

EIMs can involve numerous systems in the body, as a result of abnormal immunological response. IBD is frequently considered a systemic disorder with predominantly bowel manifestations.^3–6^ EIMs most frequently affect joints (peripheral and axial arthropathies), the skin (erythema nodosum, pyoderma gangrenosum, Sweet syndrome, aphthous stomatitis), the hepatobiliary tract (primary sclerosing cholangitis), and the eye (episcleritis, uveitis). Less frequently, EIMs also affect the lungs, the heart, the pancreas, or the vascular system. Complications such as malabsorption with consequent micronutrient deficiencies, osteoporosis, kidney stones, gallstones, and IBD drug-related side effects must be considered as complications to the disease and/or treatment.^4,7^

Multiple EIMs may occur concomitantly, and the presence of 1 EIM confers a higher risk of developing another. A Swiss IBD study showed that up to 1 quarter of EIM-affected patients tend to suffer from a combination of several EIMs,^8^ while another study showed that the chronological order of EIM appearance may be relative to timing of the IBD diagnosis, with one-fourth of the EIMs appearing prior to IBD diagnosis.^9^ A recent Danish study found that 80% of detected immune-mediated inflammatory diseases occurred before IBD diagnosis.^10^ As EIMs in IBD strongly influence quality of life and healthcare costs, adequate attention to occurrence and treatment is mandatory in affected patients. Several pathogenic mechanisms for EIMs have been proposed such as upregulation of tumor necrosis factor, aberrant lymphocyte homing, and cross-reactive antigen presentation. It still remains unclear whether EIM is a direct result of the inflammatory process in the gut or rather a consequence of a shared genetic background leading to dysfunctional immune responses to environmental stimuli.^11^

In this study, we quantified a range of EIMs found in the Danish CD and UC populations from 2003 to 2016 and compared the occurrence risks to matched populations without IBD. We also aimed to describe the time from IBD diagnosis to first experiencing an EIM and quantified the incidences that appeared before or after the diagnosis, and the occurrence of EIMs in relation to treatment with biologics. Thus, the EIMs and comorbidities included in this study were selected diagnoses based on other studies and classifications of relevant diagnoses and did not constitute an exhaustive list relevant to the study of CD and UC.^3–7,12,13^ Additionally, some diseases, for example neurological diseases, were selected for an exploratory purpose to see if a correlation was present, as at the time of analysis there was no literature published.

## MATERIALS AND METHODS

The present population-based study included all patients diagnosed with CD and UC years 2003–2015 and their EIMs and other comorbidities until end of follow-up in 2016.

### Register Data Sources

The study includes individual level data obtained from the Danish National Patient Register (NPR), which contains information on admissions and outpatient visits to hospitals for all Danish residents. Individuals were identified over time via their unique national personal identification number, Central Person Register (CPR) number, which is part of the personal information stored in the Civil Registration System.^14^ The CPR number allows for identity-secure linkage of information between national registries at an individual level. The Danish NPR includes information on all individual’s registered diagnoses.^15,16^ Diagnoses used in the current study are coded according to the 10th edition of the International Classification of Diseases (ICD-10)^17^ from 1994 and onwards.

All patients who receive biologics are treated at the hospital and can therefore be identified in the NPR when linking treatment codes and CD or UC diagnosis code. Biologic therapies available during the study period were infliximab, adalimumab, vedolizumab, and golimumab (treatment codes BOHJ18A1, BOHJ18A3, BOHJ19H4, and BOHJ18A4). The Danish Civil Registration System includes all citizens and residents with a civil personal registration number and was used for an identity-secure linkage between the national registries at an individual level.^18,19^

Additionally, as the private healthcare system constitutes less than 1% of all the healthcare provided in Denmark, only residents treated within the public healthcare system were included.

### Selection Criteria

This study was based on a population of incident patients with CD or UC in the period of 2003–2015 and followed up until 2016. Incident patients with CD (ICD-10 code K50) and UC (ICD-10 code K51) were identified in the NPR as individuals with at least 2 hospital contacts (admission, outpatient, or emergency room visit) during 2003–2015, and with at least one of the CD/UC diagnoses as a primary diagnosis. To limit the analyses to incident cases, the patients were excluded if they had a hospital contacts with CD/UC in the “wash-out” period of 1994–2002. The incidence dates for patients were defined as the first registered hospital contact with CD/UC in the period of 2003–2015. We applied the registered dates for outpatient and emergency room visits and dates for hospital admissions. Some patients with CD may have initially been misdiagnosed with UC. In case of the appearance of a later diagnosis of inflammation in the small intestines, the diagnosis was changed to CD as the correct diagnosis. Therefore, in this study, patients with this diagnostic pattern were treated as CD patients from their first hospital contact with UC. A change in diagnosis from CD to UC is considered more seldom and has not been evaluated for this study.

For each identified case, 1 control was randomly selected from the population registry and matched at January 1st in the year of IBD diagnosis on age and gender. To be considered a matched control, the control had to be alive at day of disease diagnosis. Controls were censored from the study if they developed IBD during the study period. Patients and controls were censored (ie, excluded) at death and the end of follow-up (end of 2016). In the year of death or end of follow-up, the individual was included with a weight corresponding to the fraction of the year data were available for him/her.

### EIMs and Other Comorbidities

All hospital contacts and date of first hospital registered EIM/comorbidity were identified in the NPR in the period of 1994–2015.

The selected EIMs and comorbidities for this study included 51 different diagnoses divided into eight classes: the musculoskeletal system, the skin and intestinal tract systems, the hepatopancreatobiliary system, the ocular system, the metabolic system, the renal system, neurological diagnosis, and the respiratory system. Twenty-one of the 51 EIMs/comorbidities were identified as being of special interest and analyzed separately. An overview of the EIMs and comorbidities included in the analyses and how we grouped the different diagnosis into systems can be found in Supplementary Table 1. Our experts sorted the list of suspected EIMs into the biological systems from Supplementary Table 1 to provide an overview of impact from the underlying immunological response to different types of human functions.

### Statistical Analyses

The frequency of EIMs and other comorbidities was then calculated by diagnosis class (eg, musculoskeletal system) and for all special interest diagnoses (eg, arthritis) separately of CD/UC patients and controls.

The occurrence of EIMs and comorbidities was estimated through odds ratios (ORs) and 95% confidence intervals by an unadjusted logistic regression and test for significant differences in between patient and the control groups. In addition, a separate analysis on EIMs and comorbidities in the period before and after diagnosis of IBD was conducted, as EIMs and comorbidities may occur before as well as after the CD/UC diagnosis.

For each patient and control who experienced one of the selected EIMs or comorbidities, the date of the first hospital contact with the EIM/comorbidity in question was identified in the NPR. This information allowed for an analysis of the time from CD/UC diagnosis (incidence date) to the first occurrence of an EIM or comorbidity in each diagnosis class.

All statistical analyses were conducted in SAS version 9.4 (SAS Institute Inc., Cary, NC) on Statistics Denmark’s research computers via a remote server.

### Ethical Considerations

Approval from the Danish Ethics Committee was not required by Danish law as the study only used retrospective register data. According to Danish law, access to public Danish registries is possible for authorized Danish research institutions and consultancies and after approval by the Danish Data Protection Agency. Anonymized data without contact or active participation of research subjects were used. Individuals are not identifiable and all results with fewer than 5 incidences are reported as <5 or -. The study complied with the regulations set up by the Danish Data Protection Agency (J. nr. 2014-54-0664).

## RESULTS

The characteristics of the study population are presented in Table 1. 10,302 incident patients with CD and 22,144 incident patients with UC were identified in the period of 2003–2015 and included in the analyses. The incidence of IBD in this Danish cohort has previously been published by our group along with an analysis of the societal costs of the diseases prior to diagnosis.^20^

**TABLE 1.** Characteristics of the Populations

		CD		UC	
Age at Diagnosis		N	%	N	%
0–18 years	Male	800	8%	705	3%
	Female	733	7%	717	3%
19–30 years	Male	1.225	12%	2.027	9%
	Female	1.674	16%	2.343	11%
31–40 years	Male	723	7%	1.868	8%
	Female	906	9%	1.980	9%
41–50 years	Male	601	6%	1.738	8%
	Female	727	7%	1.815	8%
51–60 years	Male	543	5%	1.603	7%
	Female	613	6%	1.652	7%
61–70 years	Male	436	4%	1.395	6%
	Female	534	5%	1.435	6%
Over 70 years	Male	282	3%	1.249	6%
	Female	505	5%	1.617	7%
Total		10,302	100%	22,144	100%
	Male	4610	45%	10,585	48%
	Female	5692	55%	11,559	52%
Total		10,302	100%	22,144	100%

The EIM/comorbidity incidence among cases and controls during 1994–2016 is presented in Table 2 for the diseases of special interest. For most EIMs/comorbidities, a significantly larger share of CD and UC patients than controls has experienced the comorbidity. In general, EIMs tend to occur at an increased prevalence in CD compared to UC patients. The risk of having at least one of the EIMs on our list of diseases of special interest as IBD patients is 27.4% for CD and 20.0% for UC, Table 3. From the full list of the 51 investigated diseases in this study, 37.8% of CD patients developed any of the EIMs/comorbidities and 30.0% of UC patients.

**TABLE 2.** Share of Population Experiencing EIMs or Other Comorbidities of Special Interest in the Period 1994–2016 Among 10,302 Patients With CD and 22,144 Patients With UC in Denmark and Their Matched Controls

	CD, % of Total Population (N = 10,302)				UC, % of Total Population (N = 22,144)			
	Patient	Control	OR	95% CI	Patient	Control	OR	95% CI
Alzheimers	0.2%	0.2%	1.05	0.57–1.94	0.4%	0.3%	1.08	0.79–1.49
Amyloidosis	0.1%	-	4.67*	1.34–16.26	0.0%	-	2.50	0.78–7.97
Ankylosing spondylitis	1.5%	0.1%	13.27*	7.37–23.88	0.7%	0.2%	3.87*	2.75–5.47
Aphthous stomatitis	0.4%	0.1%	7.19*	3.06–16.90	0.2%	0.1%	3.20*	1.79–5.72
Cirrhosis	0.6%	0.2%	3.54*	2.07–6.07	0.6%	0.2%	3.51*	2.42–5.09
CRC	1.4%	0.8%	1.74*	1.33–2.28	1.5%	1.3%	1.15	0.98–1.35
Erythema nodosum	0.8%	0.1%	9.94*	4.80–20.57	0.3%	0.0%	5.47*	2.87–10.40
Fistula anal	7.9%	0.3%	26.04*	18.46–36.73	2.1%	0.3%	6.47*	5.05–8.29
Fistula intestine	1.0%	-	53.53*	13.21–216.90	0.3%	0.1%	4.86*	2.67–8.83
Iridocyclitis (incl. uveitis/iritis)	1.3%	0.4%	2.87*	2.05–4.02	0.9%	0.4%	2.32*	1.79–3.00
Systemic lupus erythematosus (SLE)	0.2%	0.1%	1.57	0.80–3.08	0.2%	0.1%	2.04*	1.25–3.33
Osteoporosis	6.1%	2.8%	2.26*	1.96–2.60	6.4%	3.3%	2.00*	1.82–2.19
Other interstitial pulmonary diseases	0.5%	0.3%	1.75*	1.12–2.72	0.6%	0.3%	2.01*	1.50–2.68
Pancreatitis	2.7%	0.7%	3.79*	2.93–4.91	2.0%	0.7%	2.81*	2.34–3.36
Parkinson	0.3%	0.2%	1.24	0.73–2.10	0.5%	0.3%	1.58*	1.18–2.12
Primary sclerosing cholangitis	1.0%	0.1%	16.82*	7.38–38.35	1.0%	0.1%	7.55*	5.19–10.99
Psoriasis	2.2%	0.6%	3.52*	2.66–4.66	1.3%	0.6%	2.17*	1.76–2.67
Psoriatic arthritis	3.9%	0.4%	9.91*	7.21–13.64	2.6%	0.3%	8.70*	6.75–11.22
Pulmonary embolism	1.4%	0.7%	2.02*	1.52–2.67	1.8%	1.0%	1.84*	1.56–2.18
Pyoderma gangrenosum	0.5%	-	12.05*	4.34–33.43	0.4%	0.0%	16.05*	6.50–39.63
Rheumatoid arthritis	0.8%	0.4%	2.27*	1.55–3.33	1.1%	0.7%	1.59*	1.30–1.95
Any EIM	27.4%	7.3%	4.72*	4.33–5.14	20.0%	9.0%	2.53*	2.39–2.68

Note: The population includes all incident CD/UC patients in the period of 2003–2015 and their controls. CI, confidence interval.

“-” is due to a number of persons lower than 5, and numbers for such are not allowed to be shown according to the Danish Act on Processing of Personal Data.

*OR significant at the 5% level tested against the control group.

**TABLE 3.** Share of Population Experiencing EIMs or Other Comorbidities in the Period of 1994–2016 by Diagnosis Class Among 10,302 Patients With CD and 22,144 Patients With UC in Denmark and Their Matched Controls

	CD, % of Total Population (N = 10,302)				UC, % of Total Population (N = 22,144)			
	Patient	Control	OR	95% CI	Patient	Control	OR	95% CI
Skin and intestinal tract systems	15.6%	3.8%	4.71*	4.20–5.28	8.7%	4.7%	1.93*	1.78–2.08
Hepatopancreatobiliary system	10.6%	4.4%	2.57*	2.30–2.88	8.7%	5.1%	1.77*	1.64–1.91
Metabolic system	0.5%	0.4%	1.20	0.81–1.77	0.3%	0.3%	1.17	0.84–1.64
Musculoskeletal system	11.3%	3.7%	3.29*	2.92–3.71	10.2%	4.5%	2.43*	2.25–2.63
Neurological	0.5%	0.4%	1.16	0.77–1.75	0.9%	0.7%	1.32*	1.06–1.64
Ocular system	5.6%	3.0%	1.90*	1.65–2.18	4.2%	3.6%	1.19*	1.08–1.31
Renal system	2.3%	1.0%	2.29*	1.82–2.88	2.2%	1.3%	1.71*	1.47–1.98
Respiratory system	1.9%	1.0%	1.97*	1.55–2.51	2.3%	1.3%	1.88*	1.62–2.18
Any EIM	37.8%	15.4%	3.34*	3.13–3.57	30.0%	18.2%	1.92*	1.83–2.00

Note: The population includes all incident CD/UC patients in the period of 2003–2015 and their controls. CI, confidence interval.

*OR significant at the 5% level tested against the control group.

### Prevalence of EIMs/Comorbidities in Patients and Controls

In CD patients, the highest observed risks of EIMs were found among fistulas, OR 26.0 (95% 18.5–36.7) and OR 53.5 (95% 13.2–21.6), anal and intestinal fistulas, respectively, Table 2. For the 21 diseases of special interest to our group, most were found to be of significant increased risk. Following fistulas, risks were highest for primary sclerosing cholangitis, ankylosing spondylitis, pyoderma gangrenosum, and erythema nodosum, in decreasing order. For prevalence of EIMs with significant risks, the highest were anal fistula (7.9%), osteoporosis (6.1%), psoriatic arthritis (3.9%), pancreatitis (2.7%), and psoriasis (2.2%). The risk of colorectal cancer (CRC) was only significant among CD diagnosed, 1.4% at OR 3.5 (95% 1.33–2.28).

In UC patients, the highest observed risks of EIMs were found among Pyoderma gangrenosum, OR 16.1 (95% 6.5–39.6) and then psoriatic arthritis [OR 8.7 (95% 6.8–11.2)], and primary sclerosing cholangitis [OR 7.6 (95% 5.2–11.0)]. Highest prevalence with higher significant risk is seen with osteoporosis (6.4%), psoriatic arthritis (2.6%), anal fistula (2.1%), pancreatitis (2.0%), and pulmonary embolism (1.8%). Fistulas were observed to be of significant risk to UC patients for both anal [OR 6.5 (95% 5.1–8.3)] and intestinal [OR 4.9 (95% 2.7–8.8)], as well as CD patients.

The distribution of EIM/comorbidity among cases and controls independent of the timing of the diagnosis, within the 8 different organ systems during 1994–2016 is presented in Table 3. The most common EIMs/comorbidities among CD and UC patients were in skin and intestinal tract systems, the musculoskeletal system, and the hepatopancreatobiliary system (10.6%–15.6% and 8.7%–10.2% among CD and UC patients, respectively). A statistical difference between cases and controls was noted among all EIM/comorbidity classes except for neurological comorbidities of CD patients and metabolic system of both CD and UC patients. CD patients are 4.71 times more likely to have experienced an EIM/comorbidity in the skin and intestinal tract systems compared to their matched controls, Table 3. CD patients had in general a similar or higher prevalence of 1 or more diseases in the organ specified system compared to UC.

In Table 4, the populations have been stratified by whether each EIM/comorbidity was experienced before or after the patients’ IBD diagnosis, respectively. In each diagnosis class, some CD and UC patients experienced the EIM/comorbidity for the first time before their IBD diagnosis. 6.0% of all CD patients and 4.1% of all UC patients experienced EIMs/comorbidities in the skin and intestinal tract systems before their IBD diagnosis, and after IBD diagnosis, values were 9.5% and 4.6%, respectively. 5.6% of all CD patients and 4.6% of all UC patients experienced EIMs/comorbidities in the hepatopancreatobiliary system before their IBD diagnosis, similar to what was seen after diagnosis of IBD. For CD, the OR of having an EIM before or after IBD diagnosis, as compared with controls, was significant in the dermatologic, oral, hepatopancreatobiliary, musculoskeletal, ocular, renal, and respiratory systems. For UC, the risks were similar before and after UC diagnosis, apart from the nervous system where the OR was significantly higher before the diagnosis of UC, and after in the ocular system.

**TABLE 4.** Share of Population Experiencing First EIMs/Comorbidities in the Period of 1994–2016 by Class of Diagnosis, Stratified by Time of IBD Diagnosis Among 10,302 Patients With CD and 22,144 Patients With UC in Denmark and Their Matched Controls

	CD, % of Total Population (N = 10,302)				UC, % of Total Population (N = 22,144)			
	Patient	Control	OR	95% CI	Patient	Control	OR	95% CI
First EIM/comorbidity experienced before IBD diagnosis								
Skin and intestinal tract systems	6.0%	1.6%	4.05*	3.40–4.83	4.1%	2.0%	2.07*	1.85–2.32
Hepatopancreatobiliary system	5.6%	2.2%	2.56*	2.19–2.99	4.6%	2.9%	1.61*	1.45–1.78
Metabolic system	0.4%	0.4%	1.00	0.64–1.56	0.3%	0.3%	0.94	0.65–1.34
Musculoskeletal system	3.2%	1.5%	2.15*	1.77–2.60	3.8%	1.8%	2.13*	1.89–2.40
Neurological	0.2%	0.1%	1.31	0.64–2.70	0.3%	0.2%	1.60*	1.06–2.41
Ocular system	3.3%	2.0%	1.73*	1.45–2.06	2.6%	2.4%	1.08	0.96–1.22
Renal system	0.9%	0.4%	2.12*	1.48–3.04	1.0%	0.7%	1.37*	1.11–1.68
Respiratory system	0.6%	0.4%	1.54*	1.02–2.34	0.8%	0.5%	1.60*	1.26–2.03
Any EIM	15.3%	7.3%	2.54*	2.32–2.77	13.6%	9.2%	1.63*	1.54–1.73
First EIM/comorbidity experienced after IBD diagnosis								
Skin and intestinal tract systems	9.5%	2.2%	4.67*	4.03–5.41	4.6%	2.7%	1.74*	1.57–1.93
Hepatopancreatobiliary system	5.0%	2.2%	2.40*	2.05–2.82	4.1%	2.2%	1.89*	1.69–2.12
Metabolic system	0.2%	0.1%	2.29	0.94–5.56	0.1%	-	8.50*	1.97–36.80
Musculoskeletal system	8.1%	2.2%	3.90*	3.36–4.52	6.5%	2.7%	2.52*	2.28–2.78
Neurological	0.3%	0.3%	1.10	0.67–1.81	0.6%	0.5%	1.23	0.95–1.58
Ocular system	2.2%	1.1%	2.14*	1.70–2.69	1.6%	1.1%	1.41*	1.19–1.65
Renal system	1.4%	0.6%	2.39*	1.77–3.21	1.2%	0.6%	2.11*	1.71–2.60
Respiratory system	1.4%	0.6%	2.20*	1.64–2.97	1.6%	0.8%	2.05*	1.70–2.46
Any EIM	22.5%	8.1%	3.44*	3.17–3.74	16.4%	9.1%	2.03*	1.92–2.15

Note: The population includes all incident CD/UC patients in the period of 2003–2015 and their controls. CI, confidence interval.

“-” is due to a number of persons lower than 5, and numbers for such are not allowed to be shown according to the Danish Act on Processing of Personal Data.

*OR significant at the 5% level tested against the control group.

### Time From IBD Incidence to First Time Experiencing EIMs/Comorbidities

All patients who experienced EIMs/comorbidities were stratified according to time from CD/UC diagnosis to the first experience of each EIM/comorbidity (Table 5). Among patients with the most common EIMs/comorbidities (in the skin and intestinal tract systems, the musculoskeletal system, and the hepatopancreatobiliary system), 28%–52% of CD patients and 37%–53% of UC patients experienced these EIMs/comorbidities for the first time before their IBD diagnosis. Among patients who experienced EIMs/comorbidities in the metabolic system (0.5%–0.3% of all patients, Table 2), 64%–75% experienced this class of comorbidities for the first time before their IBD diagnosis. For the comorbidities not found before the IBD diagnosis, a trend is observed of experiencing them within the first year and then spread out over the follow-up years.

**TABLE 5.** Time From Diagnosis to First EIM/Comorbidity by Diagnosis Class, Among Patients With CD and UC in Denmark

	N	More Than 1 Year Before	1 Year to 1 Day Before	0–6 Months After	6–12 Months After	1–2 Years After	2–3 Years After	3–4 Years After	4–5 Years After	5–6 Years After	6–7 Years After	7–8 Years After	8–9 Years After	9–10 Years After	10+ Years After
CD patients (N)															
Skin and intestinal tract systems	1603	24%	15%	16%	6%	7%	6%	4%	4%	4%	4%	3%	2%	2%	4%
Hepatopancreatobiliary system	1092	39%	13%	10%	6%	5%	5%	4%	3%	2%	3%	3%	2%	1%	3%
Metabolic system	55	64%	-	-	-	-	-	-	-	-	0%	0%	0%	0%	0%
Musculoskeletal system	1162	22%	6%	9%	7%	11%	8%	7%	6%	5%	6%	4%	3%	2%	5%
Neurological	50	20%	14%	-	-	-	-	-	-	-	-	0%	0%	12%	-
Ocular system	572	55%	4%	5%	3%	6%	6%	5%	3%	2%	2%	2%	3%	2%	2%
Renal system	242	29%	9%	8%	5%	6%	5%	8%	6%	4%	5%	5%	2%	4%	4%
Respiratory system	197	21%	8%	12%	6%	10%	6%	6%	7%	5%	7%	-	7%	-	4%
UC patients (N)															
Skin and intestinal tract systems	1936	37%	11%	8%	4%	7%	5%	5%	4%	5%	4%	3%	2%	2%	4%
Hepatopancreatobiliary system	1935	43%	9%	9%	3%	6%	5%	4%	4%	4%	3%	3%	2%	2%	3%
Metabolic system	75	75%	-	-	-	-	-	-	0%	-	0%	-	-	0%	0%
Musculoskeletal system	2266	29%	8%	6%	5%	10%	7%	6%	6%	5%	4%	3%	3%	2%	4%
Neurological	190	18%	13%	8%	4%	6%	7%	7%	9%	6%	4%	-	5%	3%	7%
Ocular system	935	56%	7%	5%	4%	5%	4%	3%	3%	3%	2%	2%	2%	2%	2%
Renal system	484	38%	7%	7%	4%	6%	4%	6%	5%	2%	5%	3%	5%	4%	5%
Respiratory system	517	26%	7%	13%	4%	5%	7%	7%	6%	6%	3%	3%	2%	3%	6%

Note: The population includes all incident CD and UC patients in the period of 2003–2015 who experience EIM/other comorbidities.

“-” is due to a number of persons lower than 5, and numbers for such are not allowed to be shown according to the Danish Act on Processing of Personal Data. In these cases, the percentages are above 0%, resulting in the sum of the percentages shown in the corresponding rows not being 100%.

In Supplementary Table 2, an analysis similar to Tables 3 and 4 is included, but with patients on biologics indicated for CD or UC compared with incident patients not treated with biologics in the period. For patients with first EIM coming before IBD diagnosis, being treated with biologics at some point tend to present with a decreased frequency of the EIM. For patients with EIMs coming after IBD diagnosis, the tendency of prevalence seems to be the reversed for some systems, especially the skin and intestinal tract systems, and the musculoskeletal systems for both CD and UC.

## DISCUSSION

The aim of the present study was to quantify the proportion of EIMs and other comorbidities in patients diagnosed with CD and UC. As stated, the EIMs and comorbidities included in this study were selected based on other studies and classifications of relevant diagnoses, and therefore do not constitute an exhaustive list relevant to the study of CD and UC.^3–7,12,13^ The most common EIMs/comorbidities among CD and UC patients were in the skin, intestinal tract, musculoskeletal, and the hepatopancreatobiliary systems and with a significant higher risk compared to the control group. CD patients were observed to generally show higher frequencies of EIM/comorbidity relative to UC patients. Based on the risks of diseases of special interest found in IBD patients in Table 2, our results are similar to the previous findings.^3–7,12,13^

Most of the EIMs listed are found to be a risk in both CD and UC. It is evident and known that these EIMs commonly occur in the skin, joints, and eyes.^3–7^ Of note, we are showing a link between IBD and a range of interstitial pulmonary diseases (ICD-10 code J84) that may carry a significant health risk for the patients. Pulmonary manifestations of IBD present with diverse phenotypes and may cause severe symptoms and disease.^21^ Related to this, pulmonary embolism was found here to be a significant risk for both CD and UC patients compared to our control group. Pulmonary embolism has recently been under increased focus as potential adverse events of treatment with the JAK-inhibitor class of therapeutics for UC.^22^

Surprisingly, we found that UC patients also experienced intestinal and anal fistulas which most likely could be explained as complications to previous surgery where CD can appear in a pouch in an UC patient after resection. This is in line with observations that a complication to ileal pouch anal anastomosis can be the de novo appearance of CD.^23,24^ It was not possible to correct for that, as UC patients with a subsequent CD diagnosis was corrected to be a CD patient back from incidence.

It is well established that IBD diagnosis result in increased risk of developing CRC, as high risk as 2–6 times the background population,^25–28^ however, in the present study an OR of approximately 1.7 was observed in CD patients, but surprisingly not significantly higher OR for UC patients vs controls. There are 2 possible explanations to this finding. First, CRC might be found as a significant risk with a larger control group than the 1:1 matching used here, as a nominal trend is observed, and second, several studies report that the CRC prevalence in the IBD population is decreasing over time, including an investigation of the Danish patients,^29–31^ and this study presents cohort of 2003–2015. In Denmark, the explanation for this may be that a relatively early use of surgical intervention will prevent CRC from developing at a later stage. Compared to controls, UC patients were significantly more likely to be diagnosed with Parkinson disease, similar to previously reported studies from Denmark and Sweden.^32,33^ Interestingly, when analyzed with Alzheimer diseases as diseases in the neurological system, we found that the diseases were only significantly associated with UC if the neurodisease was diagnosed before UC. This has not been discussed before and warrants further investigation.

The final difference in risks between CD and UC is for systemic lupus erythematosus that we only found to be significant in UC patients. This risk was not found in a recent Danish study.^10^ Like with many of the results here, our findings show that there appears to be an increased risk in patients with UC developing systemic lupus erythematosus. However, as this is a register-based population study, we are unable to make a causal link to this finding or others.

In agreement with previous studies, patients were found to have a risk of experiencing their first EIMs/comorbidities prior to their IBD diagnosis.^7,9,10^ Further, IBD patients were likely diagnosed with the same EIMs before and after IBD diagnosis. This study is reporting 37.8% risk of any EIMs/comorbidity for CD and 30.0% for UC, but from our full list of investigated diseases.

In addition, the patients were stratified by whether or not they received biologic treatment in the period 2003–2016, to compare the risks of developing EIMs/comorbidities, Supplementary Table 2. There were significant risk differences between patients who received biological treatment and patients who did not in developing EIM/comorbidity classes, but the results should be interpreted with caution. This study is observational and not causal. Patients treated with biologics have moderate to severely active disease as per medication indication, which likely also correlate with higher risk of comorbidities. Also, some biologics used in CD or UC treatment are also indicated for a range of other diseases. Therefore, here the biologics could be prescribed for other primary diagnosis than CD and UC in the present study. Some disease systems had an increase in risk when the patients had been treated with biologics, as mentioned in the results, especially for CD, but diseases in the respiratory system in CD patients showed a decrease risk with biological treatments. This study pools all biological treatments, which in the years of the cohort consists of a vast majority of anti-tumor necrosis factor-α medications, to a very small extent an anti-integrin biologic treatment. In contrast to these observations, a recent Danish study finds that the treatment of infliximab decreases the risk of developing immune-mediated inflammatory diseases overall after diagnosis of both CD and UC, but in a considerably shorter list of diseases.^10^

A diagnostic delay of IBD is well recognized and the median time from first symptom to diagnosis may be between 2 and 9 months form recent publications, with CD tending to be longer than UC.^34–37^ Is has also recently been reported how underlying factors like biomarkers and healthcare utility may indicate that patients are on a path toward developing IBD before clinical symptoms appear.^20,38^ The shared risks of these EIMs in patients when looking before the IBD diagnosis points toward a common etiology where the gastroenterology units may be involved earlier. Conversely, IBD patients have increased risk at developing other diseases, which may need other specialties in the early phase. This underlines the importance of a multidisciplinary care team and may have implications on the type of medicine being offered when taking the underlying etiology into account. More research within this scope is warranted.

### Strengths and Limitations

This study is based on national registers, which prevents selection and information bias, as all Danish residents are included, the data are prospectively collected, and the quality of data is generally considered to be high. This also means that the generalizability of the results is high, as the study covers all incident cases of CD and UC in Denmark between 2003 and 2015. Furthermore, by using a wash-out period we ensured that the selected population of CD and UC cases were in fact incident patients. As CD and UC are severe chronic diseases, patients will be in contact with hospitals for their disease for the rest of their life, however at different frequencies. If the disease is well treated or in remission, the patient may not be in contact with the hospital in that period. Therefore, we used a long wash-out period of 9 years to ensure that we found only incident patients in the study period.

This study also has some limitations. The study relies on registry collected information. Furthermore, there is a risk of misclassification, as identifying CD and UC patients as well as the EIMs/comorbidities, we relied on the accuracy of the ICD-10 coding in the NPR. The reliability and validity of the diagnosis registration in the NPR have been assessed to be good, however there have not been any studies that explicitly validate the registration of CD and UC diagnosis codes.^15,39,40^ The study aimed to prevent the potential of confounding by direct matching on age and gender, however as in all observational studies without random allocation to the exposure groups, ie, individuals with and without IBD in the present study, unmeasured confounding may still affect the validity. We are aware that the severity of CD and UC may influence the risk of development of EIMs or other comorbidities, but measures of disease state were not included in the data from the Danish registry. Further investigations incorporating registries with IBD databases would be beneficial.

## CONCLUSIONS

This study provides population-based evidence that EIMs in CD and UC patients have a similar risk of occurrence, pre- and post-IBD diagnosis, but may also show some interesting differences between the IBDs. The shared risks of these EIMs points toward a common etiology and the importance of multidisciplinary care. The findings may indicate a diagnostic delay of CD and UC, and the occurrence of known EIMs should prompt physicians to look for underlying IBD, especially if abdominal issues are observed.

## Supplementary Material

otaa070_suppl_Supplementary_Tables

## Data Availability

The data underlying this article were provided by Statistics Denmark by permission. The data underlying this article cannot be shared publicly due to ethical reasons.

## Notes

Funding: This work was supported by Janssen Pharmaceuticals. Data collection was carried out by incentive, with financial support from the funder. The funder provided support in the form of salaries for authors K.V., S.A., A.B., and T.R.J.

Author Contribution: The authors have contributed as follows: K.V., T.R.J., N.Q., and P.M. contributed to the concept and design of the study, review and interpretation of the data, drafting and critical review of the manuscript, and approval of the final draft. S.A. and A.B. contributed to the review and interpretation of the data, drafting and critical review of the manuscript, and approval of the final draft. A.N. contributed to the concept and design of the study, data acquisition, extraction and analysis, review and interpretation of the data, drafting and critical review of the manuscript, and approval of the final draft.
