# Supplementary material for: Extraintestinal Manifestations and Other Comorbidities in Ulcerative Colitis and Crohn Disease: A Danish Nationwide Registry Study 2003–2016
Source: Crohns Colitis 360. 2020 Aug 25;2(3):otaa070. doi: 10.1093/crocol/otaa070 (PMC9802257; doi:10.1093/crocol/otaa070)
Supplement: otaa070_suppl_Supplementary_Tables [file otaa070_suppl_supplementary_tables.docx]

Appendix

Supplementary Table 1. List of EIMs and other comorbidities included in the analysis

| **Diagnosis class** | **Subgroup** | **EIM/comorbidity** | **ICD-10 code** |
| --- | --- | --- | --- |
| Musculoskeletal system | Arthritis | *Psoriatic arthritis | M07 |
|  |  | *Rheumatoid arthritis | M05 |
|  |  | *Ankylosing spondylitis | M45 |
|  |  | * Systemic lupus  Erythematosus (SLE) | M32 |
|  |  | Sacroiliitis | M43.2 |
|  | Hypertrophic  osteoarthropathy | Clubbing | R68.3 |
|  |  | Periostitis | M90.1 |
|  | Misc. Manifestations | *Osteoporosis | M80-M82 |
|  |  | Aseptic necrosis | M87 |
|  |  | Polymyositis | M33.2 |
| Skin and intestinal tract systems | Reactive lesions | *Erythema nodosum | L52 |
|  |  | *Pyoderma gangrenosum | L88 |
|  |  | *Aphthous stomatitis | K12.0 |
|  |  | Necrotizing vasculitis | M30-M31 |
|  | Specific lesions | *Fistula anal | K603-605 |
|  |  | *Fistula intestine | N321, N832A, K63.2 C, D, F, G |
|  | Nutritional deficiencies | Acrodermatitis enteropathica | E83.2 |
|  |  | Thrombocytopenic purpura | M31.1 |
|  |  | Glossitis | K14.0 |
|  |  | Hair loss | L65.9 |
|  |  | Brittle nails | L60.3 |
|  | Associated diseases | Vitiligo | L80 |
|  |  | *Psoriasis | L40 |
|  |  | *Amyloidosis | E85 |
|  |  | Sweet's syndrome | L98.2 |
|  | Malignancies | Melanoma | C43 |
|  |  | Non-melanoma skin cancer | C44 |
|  |  | *Colorectal cancer | C18-C21 |
| Hepatopancreatobiliary system |  | *Primary sclerosing cholangitis | K83.0 |
|  |  | Bile duct carcinoma (Cholangiocarcinoma) | C22.1 |
|  |  | *Pancreatitis | K85, K86.1 |
|  | Associated inflammation | Autoimmune chronic active hepatitis | K75.9 |
|  |  | Portal fibrosis | K74.0 |
|  |  | *Cirrhosis | K74 |
|  |  | Granulomatous disease | D71 |
|  | Metabolic manifestations | Fatty liver | K76.0 |
|  |  | Gallstones/cholelithiasis | K80 |
| Ocular system |  | *Iridocyclitis (incl. uveitis/iritis) | H20 |
|  |  | Scleritis/episcleritis/scleromalacia | H15 |
|  |  | Corneal ulcers | H16.0 |
|  |  | Retinal vascular disease | H34, H35.0 |
|  |  | Conjunctivitis | H10 |
|  |  | Blepharitis | H01.0 |
|  |  | Cataract | H28.8 |
| Metabolic system |  | Growth retardation | R62.0 |
|  |  | Delayed sexual maturation | E30 |
| Renal system |  | Calcium oxalate stones | N20.0 |
| Neurological |  | *Parkinson | G20 |
|  |  | *Alzheimers | Q30 |
| Respiratory system |  | *Other interstitial pulmonary diseases | J84 |
|  |  | *Pulmonary embolism | I26 |

Note: EIMs and other comorbidities marked with “*” are of special interest to this study and analysed seperately.

Supplementary Table 2. Share of patients with or without biological treatment experiencing EIMs/comorbidities in the period 1994-2016, stratified on time of first EIM/comorbidity by diagnosis class

|  | CD, % of patient population | | | | UC, % of patient population | | | |  |
| --- | --- | --- | --- | --- | --- | --- | --- | --- | --- |
|  | No biological treatment^1^ | Biological treatment^2^ | Odds ratio | 95%CI | No biological treatment^1^ | Biological treatment^2^ | Odds ratio | 95%CI | |
| **First EIM/comorbidity experienced before IBD diagnosis** |  |  |  |  |  |  |  |  | |
| Skin and intestinal tract systems | 6.0% | 6.2% | 0.97 | 0.81-1.15 | 4.1% | 4.4% | 0.94 | 0.77-1.16 | |
| Hepatopancreatobiliary system | 6.2% | 4.0% | 1.57* | 1.28-1.93 | 4.8% | 3.0% | 1.66* | 1.30-2.11 | |
| Metabolic system | 0.2% | 0.8% | 0.28* | 0.15-0.52 | 0.3% | 0.3% | 0.80 | 0.38-1.68 | |
| Musculoskeletal system | 3.6% | 2.1% | 1.73* | 1.31-2.29 | 3.9% | 2.8% | 1.41* | 1.10-1.81 | |
| Neurological | 0.2% | - | 1.86 | 0.54-6.49 | 0.3% | - | 7.41* | 1.03-53.55 | |
| Ocular system | 3.5% | 2.9% | 1.20 | 0.93-1.53 | 2.6% | 3.1% | 0.84 | 0.66-1.07 | |
| Renal system | 1.0% | 0.7% | 1.29 | 0.80-2.09 | 1.0% | 0.6% | 1.58 | 0.95-2.64 | |
| Respiratory system | 0.6% | 0.3% | 1.88 | 0.95-3.73 | 0.8% | 0.3% | 2.63* | 1.29-5.34 | |
| **First EIM/comorbidity experienced after IBD diagnosis** |  |  |  |  |  |  |  |  | |
| Skin and intestinal tract systems | 6.7% | 16.7% | 0.36* | 0.31-0.41 | 4.5% | 5.4% | 0.83* | 0.69-1.00 | |
| Hepatopancreatobiliary system | 4.6% | 6.1% | 0.76* | 0.63-0.91 | 4.1% | 4.2% | 0.97 | 0.79-1.20 | |
| Metabolic system | 0.2% | - | 1.73 | 0.49-6.08 | 0.1% | - | 0.96 | 0.22-4.18 | |
| Musculoskeletal system | 6.8% | 11.5% | 0.56* | 0.48-0.65 | 6.2% | 8.6% | 0.70* | 0.60-0.81 | |
| Neurological | 0.4% | - | 6.21* | 1.49-25.95 | 0.7% | - | 5.47* | 1.74-17.18 | |
| Ocular system | 2.1% | 2.4% | 0.87 | 0.66-1.16 | 1.5% | 2.0% | 0.78 | 0.58-1.06 | |
| Renal system | 1.5% | 1.4% | 1.02 | 0.71-1.46 | 1.2% | 1.4% | 0.83 | 0.58-1.18 | |
| Respiratory system | 1.6% | 0.7% | 2.16* | 1.37-3.41 | 1.6% | 1.6% | 1.00 | 0.72-1.40 | |

Note: The population includes all incident CD/UC patients in the period of 2003-2015

“-“ is due to a number of persons lower than 5, and numbers for such are not allowed to be shown according to the Danish Act on Processing of Personal Data

*: Odds ratio significant at the 5% level tested against the control group.

^1^: The patient have not received treatment with biologics in the period of 2003-2016.

^2^: The patient have received treatment with biologics at some point in the period of 2003-2016.
